# Glove and mitten protection in extreme cold weather: an Antarctic study

**DOI:** 10.3402/ijch.v75.33564

**Published:** 2016-11-10

**Authors:** Kenneth V. Iserson

**Affiliations:** Department of Emergency Medicine, The University of Arizona, Tucson, AZ, USA

**Keywords:** clothing, hand warmth, Antarctica, hand protection, hand comfort, cold injury prevention, protective gear

## Abstract

**Background:**

Myths, misconceptions and a general lack of information surround the use of gloves and mittens in extreme cold environments.

**Objective:**

This study assessed how well an assortment of gloves and mittens performed in a very cold environment.

**Methods:**

A convenience sample of gloves and mittens were tested in Antarctica during the winter of 2016 using a calibrated thermometer (range: −148°F to +158°F/−100°C to +70°C) three times over a 0.5-mile distance (~20 minutes). A small sensor on a 10-foot-long cable was taped to the radial surface of the distal small finger on the non-dominant hand. The tested clothing was donned over the probe, the maximum temperature inside the glove/mitten was established near a building exit (ambient temperature approximately 54°F/12°C), and the building was exited, initiating the test. The hand was kept immobile during the test. Some non-heated gloves were tested with chemical heat warmers placed over the volar or dorsal wrist.

**Results:**

The highest starting (96°F/36°C) and ending (82°F/28°C) temperatures were with electrically heated gloves. The lowest starting temperature was with electrically heated gloves with the power off (63°F/17°C). Non-heated gloves with an inserted chemical hand warmer had the lowest minimum temperature (33°F/1°C). Maximum temperatures for gloves/mittens did not correlate well with their minimum temperature.

**Conclusions:**

Coverings that maintained finger temperatures within a comfortable and safe range (at or above 59°F/15°C) included the heated gloves and mittens (including some with the power off) and mittens with liners. Mittens without liners (shell) generally performed better than unheated gloves. Better results generally paralleled the item's cost. Inserting chemical heat warmers at the wrist increased heat loss, possibly through the exposed area around the warmer.

In very-low-temperature environments, cold sensation and injury most commonly affect hands, feet and exposed areas of the head. A variety of protective gear exists to limit cold exposure to these areas. No perfect cold-protective gear exists for extremely low temperatures, and users often find it difficult to determine which equipment to choose. The varying materials, measurement systems and manufacturers’ claims can be confusing and frustrating. Users often must rely on word-of-mouth from other users to select appropriate clothing.

Winter in Antarctica provided an opportunity to gather data on how well various hand protection systems (i.e. mittens, gloves and liners) performed in a very-low-temperature environment. Based on experience and on the published literature, the expectation was that hand coverings retaining the most warmth would be, in descending order, electrically heated mittens, heated gloves, unheated mittens with liners, unheated mittens, unheated gloves with liners, unheated gloves and liners. This study was done to discover whether this commonly accepted paradigm is correct, and to go beyond the subjective assessments upon which most users must rely.

## Methods

This study was performed at McMurdo Station, Antarctica (77.8419°S, 166.6863°E), during the winter of 2016 (mid-February through mid-August). The mitten or glove and liner combinations tested included those personally owned as well as a convenience sample of work gloves and “extreme cold weather” gear supplied to personnel based in McMurdo Station (USA) and Scott Base (NZ), who, in turn, submitted them for testing.

Each test period was the time needed to traverse 0.5 miles through the town and along the heights adjacent to the normally windy sea ice, which is generally about 20 minutes. Each combination of mitten or glove and liners was tested at the same time on three different days to confirm result accuracy. If the difference between the test results was >1°F (0.6°C), another test was performed and the outlier discarded. The ambient and wind chill temperatures were obtained from the Station's weather service. While weather conditions, including temperature and wind chill, varied between tests and often during the test periods, ambient temperatures during all tests were ≤−10°F (−23°C).

**Fig. 1:**
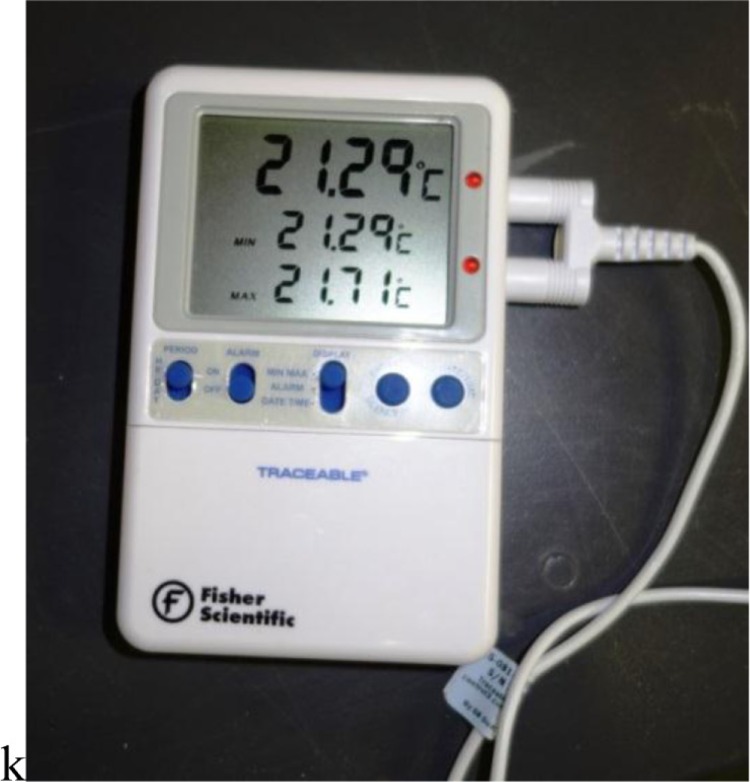
Fisher Scientific™ Traceable™ Platinum High-Accuracy Refrigerator/Freezer Thermometer with Probe, catalogue number 15-081-109.

The author was the subject for all tests and wore the same body-protective clothing on each test. Temperatures were measured using a calibrated Fisher Scientific™ Traceable™ Platinum High-Accuracy Refrigerator/Freezer Thermometer with Probe (catalogue number 15-081-109; Fig. 1), with a range of −148.00°F to +158°F (−100.00°C to +70°C). The included sensor was a bullet-sized, waterproof temperature probe attached to a 10-foot-long flexible cable. The probe was taped to the radial (inner) surface of the distal small finger on the non-dominant hand. The gloves to be tested were then donned over the probe and maximum temperature was passively achieved by standing in a building-exit anteroom (ambient temperature averaged 54°F/12°C); at that point, the building was exited and the test begun. The hand was kept immobile during the test.

Some gloves were also tested with unexpired chemical heat warmers placed over the volar or dorsal wrist. This was done to test a commonly held belief among Station personnel that this measure keeps hands warmer, even with poor-quality gloves.

The maximum and minimum temperatures for each glove test were recorded and stored in the thermometer. Heated mittens/gloves were tested with their batteries fully charged. To avoid the large variation in battery life at low temperatures, battery life was tested at 72°F (22°C).

No institutional review board approval was required, since the author was the sole subject.

## Results

The table contains the test results of each hand protection combination (Table 1). Since in cold environments, hand comfort over time relates most to a glove/mitten's minimum temperature, the table lists, from highest to the lowest, the average minimum temperature achieved during the three tests for each hand protection combination. The bottom 14 combinations are gloves/mittens/liners that allowed the hands to become colder than the minimum recommended “Winter” Comfort Zone (1) and the temperature causing pain in the palm of the hand when touching a cold object (2).

The wind chill during all tests ranged from −20°F to −35°F (−29°C to −37°C). The length of time of each test period also varied slightly, ranging from 14 to 17 minutes. Indoor battery life for both the Volt and Outdoor Research products at maximal power was between 3.5 and 4 hours. Performance of fine motor tasks was difficult when wearing all tested mittens and gloves. Wearing only glove liners allowed the user to perform fine motor tasks, but only for a short while before the fingers got too cold.

**Table 1:** Ordered test results: highest to lowest minimum temperature^a^

Glove/mitten/liner^b^	Minimum temp during test: °f (°c)	Maximum temp when beginning test: °f (°c)	Heat loss during test: °f (°c)	Retail cost: US$
Combinations that kept hands above the safe “Winter” Comfort Zone (1) and the temperature causing pain in the palm of the hand when touching a cold object (2)				
Outdoor Research heated gloves^A^ (pre-heated max)	82 (28)	96 (36)	14 (8)	245
Volt heated mittens^B^ (pre-heated 4 bars)	81 (27)	93 (34)	12 (7)	190
Marmot 8000 Meter Mitt/Marmot liners^C^	80 (27)	92 (33)	12 (6)	275^c^
Outdoor Research Alti Mitts^D^/Mountain Hardwear liner^E^	74 (23)	84 (29)	10 (6)	279
Outdoor Research heated gloves^A^ (battery off)	70 (21)	93 (34)	23 (13)	245
Outdoor Research Alti Mitts and liner^F^	67 (13)	80 (27)	13 (7)	239
Volt heated gloves^G^ (pre-heated 4 bars)	66 (18)	95 (35)	29 (17)	220
Volt heated mittens^B^ (battery off)	59 (15)	88 (31)	29 (16)	190
Generic mittens^H^/possum liner^I^	59 (15)	75 (24)	16 (9)	55
Combinations that failed to keep hands above the safe “Winter” Comfort Zone (1) and the temperature causing pain in the palm of the hand when touching a cold object (2)				
Outdoor Research Alti Mitts^F^/no liner	56 (13)	74 (23)	18 (10)	199
Generic mittens^H^/no liner	52 (11)	78 (26)	26 (15)	75
Kinco gloves^J^/no hand warmer inserted	50 (10)	82 (28)	32 (18)	21^d^
Pro-Val CE gloves^K^/hand warmer at dorsal wrist^e^	50 (10)	76 (24)	26 (14)	5^d^
Marmot 8000 Meter Mitt^C^ shell only/no liner	48 (9)	82 (28)	34 (19)	275^c^
Pro-Val CE gloves^K^/hand warmer at volar wrist^e^	48 (9)	90 (32)	42 (23)	5^d^
Carhartt gloves^L^	46 (8)	82 (28)	36 (20)	39^d^
Mountain Hardwear glove liner^E^	43 (6)	75 (24)	32 (18)	40
Mechanix Wear Cold Weather gloves^M^	42 (6)	82 (28)	40 (22)	22^d^
Volt heated gloves^G^ (battery off)	41 (5)	63 (17)	22 (12)	220
Pro-Val CE gloves^K^/no hand warmer	39 (4)	81 (27)	42 (23)	5^d^
Palamino thinsulate gloves^N^	34 (1)	86 (30)	52 (29)	22^d^
Firm grip all-purpose winter gloves^O^	33 (1)	87 (31)	54 (31)	10
Kinco gloves^J^/hand warmer at volar wrist^e^	33 (1)	82 (28)	49 (27)	21^d^

a Numbers rounded to the nearest significant digit;

b Capital letters refer to list of mitten/glove/liner combinations;

c issued to the Scott Base (NZ) Search and Rescue (SAR) team;

d issued by United States Antarctic Program (USAP);

e all hand warmers used were USAP-issued air-activated chemical Grabber^®^ Hand Warmers (Kobayashi Consumer Product, China).

A Outdoor Research Lucent Heated Gloves™; GORE-TEX^®^ inserts, EnduraLoft™ insulation.

B Volt Heated Mittens: Maxima 7v™ Nylon Heated Snow Mitts; Model GL-7V-MXMT.

C Marmot 8000 Meter Mitt: Sold as shell-liner combination. Primaloft^®^ Insulation, 700+ Fill Power Goose Down; GORE-TEX^®^Mitt Insert.

D Outdoor Research Alti Mitts™/Mountain Hardwear Liner: A typical combination.

E Mountain Hardwear Glove Liner: Power Stretch^®^ Stimulus™ Glove; Model 1552721090/OM6248-090. Polartec^®^ Power Stretch^®^.

F Outdoor Research Alti Mitts™/Alpine with inserts; Model 71892. GORE-TEX^®^, leather palms (Pittards Armortan™), Primaloft One™ insulation.

G Volt heated gloves: Titan Men's 7v™ Leather Heated Gloves; Model GL-7V-TNM-L.

**Fig. 2:**
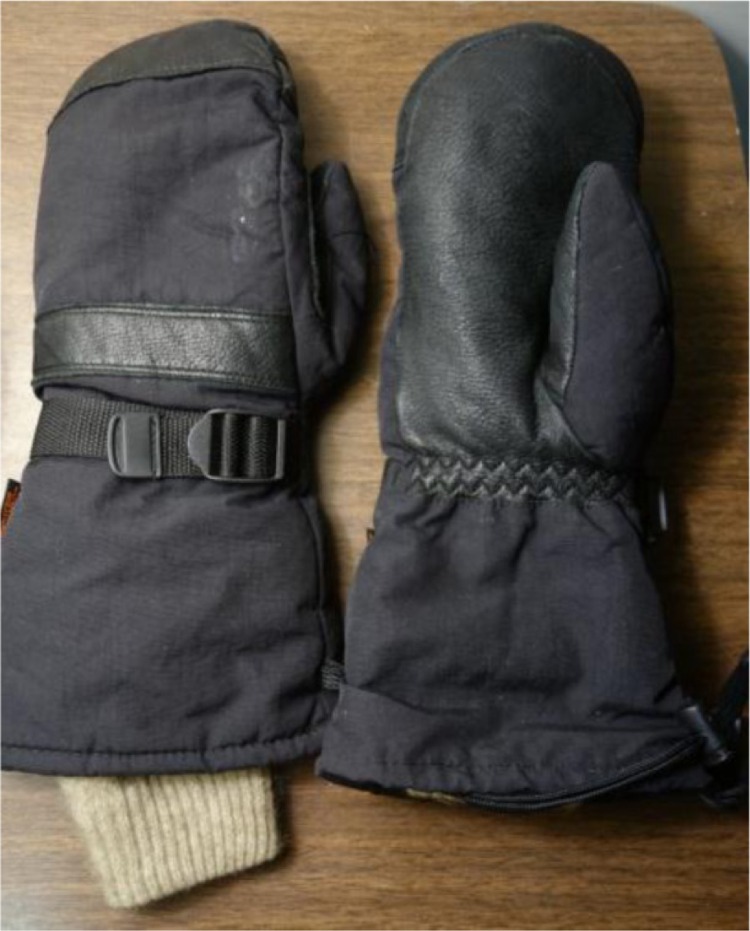
Generic mittens with possum fur liner; unknown brand.

H Generic mitten (unknown brand) with Ski-Dri2 Thinsulate™ Supreme insulation (Fig. 2). (Mitten and liner prices provided by owner.)

I Possum fur liner: generic from New Zealand.

J Kinco Gloves: HeatKeep^®^ insulation.

K Pro-Val CE Gloves: Model 41515/3242.

L Carhartt Gloves: A505/BlkBLY; RN#56385.

M Mechanix Wear Cold Weather Gloves: PUC-901-13;RN83381;CA#34971.

N Thermal Insulated Palomino™: 100G Thinsulate™ Insulation.

O Firm Grip All-Purpose Winter Gloves: Model 2185.

## Discussion

In extreme cold environments, personal and group experiences with gloves and mittens, as well as cost factors, guide individuals’ protective clothing choices. This study was done to provide an alternative method for groups and individuals to apply when making future cold-weather clothing selections.

### Cold exposure

Cold exposure can reduce blood flow to the hands, decrease manual dexterity and cause injury (3). Arctic researchers recently found that at an ambient temperature of 23°F (−5°C) both fine and gross manual dexterity was reduced, resulting in “decreased performance and increased risk of mistakes.” (4)

In very cold environments, cold hands constitute a common, and often major, medical problem (3,5,6). One Finnish study of 2,555 young people demonstrated that only the head is more prone to frostbite than are the hands (6). Likewise, studies of civilian workers in Norway demonstrated that fingers and hands are most vulnerable to cooling, causing a degradation in manual performance (4). Frostbite in fingers develops after about 15 minutes of exposure to −0.4°F to −9.4°F (−18°C to −23°C) (7).

Multiple external factors determine the effect of cold weather on, and risk for, humans in addition to their clothing. These include the environment (i.e. air temperature, wind-speed, precipitation and radiation), activity level, physical contact (e.g. with cold objects) and psychosocial mindset (e.g. impact of darkness on mood) (4).

Multiple individual factors also play a role, including health, fitness, acclimatization and level of experience (4). One assessment tool, using cold-induced vasodilation (CIVD) in the finger tips (8), has demonstrated changes in peripheral blood flow and skin temperatures in black people, in females, at high altitudes, with increasing age, with specific diets (increased Vitamin C, protein and salt) and when under stress (9). This is probably caused by a sudden decrease in neurotransmitter release from the sympathetic nerves to the arteriovenous anastomoses that are specific thermoregulatory organs that regulate blood flow (9).

### Glove-testing methods

This study used a single human subject to avoid the variability among different testers (10), a calibrated digital temperature probe rather than a subjective cold–hot scale (11,12) and a natural environment rather than a laboratory setting with manikins, chambers, thermography, thermal resistance or similar methods used in previous tests (10,12). The temperature probe was placed on the distal pulp of the non-dominant fifth finger, since prior studies showed that this site was suitable and that the non-dominant hand was colder than the dominant hand after acute cold exposure (13,14).

### Glove design, fabrics and heated gloves

Appropriate clothing helps retain the body's warmth by limiting the heat exchange between the body and the environment. The amount of heat loss that clothing prevents depends on its “thermal properties (insulation, evaporative-, wind- and water resistance), design and construction (weight, fibers and fabrics, ergonomics)” (4). Warming the torso, even with the extremities exposed to freezing temperatures, maintains finger and toe comfort for an extended period of time (15).

Disagreements about what constituted optimal extreme cold-weather clothing were evident among early Antarctic explorers. During his attempt to reach the South Pole in 1911, Roald Amundsen used reindeer skins with seal fur as his team's primary clothing insulators. Capt. Robert Falcon Scott, however, used cotton and wool, a combination that has a lower insulation-to-mass ratio and increases the clothing's weight, bulkiness, friction and stiffness – factors that may have contributed to his team's disastrous failure in 1912. Those venturing into extremely cold environments now can choose from multiple modern lightweight fabrics and combinations (2,4).

To control hand temperature and humidity in varying conditions, natural fibers like wool that have excellent insulation properties and high moisture-absorbing capacity are often used for the inner layer. Hydrophobic synthetic fibers are then used to move moist air to the next fabric layer (4). In practice, many people working in cold environments layer their hand coverings, using a thin polypropylene glove liner under a thick pile or wool mitten, covered by an outer windproof/waterproof layer of leather or Gore-Tex^®^ (16).

Mittens, which have long been recommended over gloves for protection from cold, limit finger dexterity (16,17). Yet, except for the thinnest liners, all the tested hand coverings made manipulating gear, taking photos, starting fires, holding keys, preparing food and eating difficult or impossible without removing at least the layer covering the thin liner. After removing hand coverings for these tasks, this study found that only battery-powered gloves and mittens generated enough energy to rewarm hands quickly, eliminating the need to wait for the body to generate heat to those peripheral areas.

As in this study, the US Army cold-weather researchers found that, in general, heated hand coverings, although they were less bulky and had less intrinsic insulation, maintained higher finger temperatures (when turned on) than the bulkier unheated gloves “when the heated gloves functioned properly” (12). In this study, the purpose of testing heated gloves without battery activation was to determine how well they would function if the batteries failed. Most commonly, the batteries were affected by the extreme cold, and held their charge for a shorter period than they would under less stressful circumstances and during indoor testing. Of note, none of the tested battery connections consistently remained attached when used in a vibrating vehicle, such as the tracked passenger vans used for travel across the ice, causing the detached gloves/mittens to turn off.

### Results compared to pre-test hypothesis

This study's results varied slightly from pre-test expectations. Coverings that maintained finger temperatures in a comfortable and safe range (59°F/15°C) included the heated gloves and mittens (including some with the power off) and mittens with liners. Mittens without liners (just the shell) generally performed better than unheated gloves. The only glove liner tested separately performed better than some unheated gloves. Better results generally paralleled the item's cost.

Some non-heated gloves were tested with dorsal and volar chemical heat warmers, since many Station personnel believed that inserting warmers over the dorsal or volar wrist elevates hand temperatures. In fact, the opposite occurred. Although their initial maximum temperatures were often higher, the heat packets’ bulk created gaps at the wrist, causing more rapid hand cooling than if no heat warmers had been used.

After testing, we found that we could not identify a brand or manufacturer for the “generic mittens” lent to us for this study by a colleague, which we had coupled with a generic possum fur liner (commonly available in New Zealand). The results for this item were included to demonstrate that there is room for improvement in glove and mitten design at a reasonable price. The factors observable in this product were that it was a mitten; had a breathable, water-resistant outer shell; had a Ski-Dri2 Thinsulate lining; and was supplemented by an inexpensive possum fur glove liner (Fig. 2).

## Limitations

In this study, the supply of gloves and mittens was limited to a convenience sample of gear available among team members at McMurdo Station and Scott Base. This ad hoc sampling method led to the inability to identify the “generic” brand of the mitten that was the least expensive and still acceptable combination; the accompanying possum fur liner gloves are often not sold under a brand name in New Zealand, where they were purchased.

The testing methods used in this study varied from those used by glove manufacturers and reported to the public. The ambient and wind chill temperatures varied slightly, as would be expected in non-laboratory conditions. No formal tests were made of durability or of the ability to perform fine motor tasks.

## Conclusions

In this study, coverings that maintained finger temperatures within a comfortable and safe range (≥59°F/15°C) included the heated gloves and mittens (including some with the power off) and mittens with liners. Mittens without liners (just the shell) generally performed better than unheated gloves. The only glove liner, tested separately, performed better than some unheated gloves. Better results generally paralleled the item's cost. Contrary to popular myth, placing chemical warming packs at the wrist decreases effectiveness, possibly through exposing the skin in the area.

## Financial/Material Support Statement

Except for the Outdoor Research Alti Mitts and the Mountain Hardwear liners, all gloves, mittens and liners for this study were on loan from either the company (Volt heated mittens and gloves) or individuals.
